# Analysing the effect of resilience and perceived social environment on university students' intention to start sustainable ventures

**DOI:** 10.1371/journal.pone.0301178

**Published:** 2024-04-16

**Authors:** Gustavo Barrera-Verdugo, Jaime Cadena-Echverría, Daniel Durán-Sandoval, Antonio Villarroel-Villarroel

**Affiliations:** 1 Faculty of Engineering and Business, Universidad de Las Américas, Providencia, Santiago, Chile; 2 Escuela Politécnica Nacional, Facultad de Ciencias Administrativas, Quito, Ecuador; National Taichung University of Science and Technology, TAIWAN

## Abstract

The literature mostly has addressed the determinants of entrepreneurial intention in general and social entrepreneurship, without focusing specifically on sustainable entrepreneurial intention despite it has become highly relevant in addressing environmental degradation and social challenges. This study aims to contributes to the understanding of psychological and social factors that influence sustainable business, evaluating the effect of resilience, subjective norms, and perceived social support on sustainable entrepreneurial intention. We analysed the online survey responses of 433 students from Chile and Ecuador enrolled in business and engineering programs using Partial Least Squares Structural Equation Models (PLS-SEM), moreover, a multi-group analysis (MGA) has been conducted to assess gender differences. The findings have supported the positive effect of psychological resilience and subjective norms on perceived social support, besides, an important effect of social support on sustainable entrepreneurial intention was found. These results offer new evidence regarding the significance of the social environment, support networks, and resilience of university students in fostering the establishment of sustainable businesses with a focus on social well-being and environmental protection in Latin America. This is particularly pertinent as the countries in this region are grappling with social and environmental challenges that could be addressed through sustainable entrepreneurship.

## Introduction

Entrepreneurship is a human endeavour aimed at identifying, transforming, and profiting from problems whose solutions generate economic and social value [1]. Evolving societal demands, climate change, and the ecological transition are driving companies to modify their practices in order to incorporate social and environmental concerns. However, certain enterprises are inherently sustainable and thrive within the current economy, led by sustainable entrepreneurs who perceive opportunities within social and environmental issues [2]. In this context, sustainable entrepreneurship is defined as business practices that balance profitability with contributions to people and the planet [3]. Specifically, it involves the identification of new business opportunities that result in sustainable products, production methods, or ways of organizing business processes [4].

Sustainable entrepreneurship plays a pivotal role in national economies as it enables the implementation of various environmentally friendly practices at the enterprise level, thereby fostering conducive conditions for sustainable economic development [5, 6]. Furthermore, sustainable businesses exert a positive influence on curbing the utilization of natural resources, minimizing waste generation, and reducing emissions [7], while also contributing to the advancement of sustainable development by addressing key United Nations Sustainable Development Goals [8], including the reduction of inequalities, climate action, and the promotion of responsible consumption. Consequently, the support for the advancement of sustainable entrepreneurship has become imperative in light of the challenges confronting businesses and societies at large, particularly those associated with climate change [9].

A major variable that promotes the performance of an action is the intention to perform such action, which can be understood as a mental state that powerfully predicts and explains human thinking and behavior [10]. In the field of entrepreneurship, entrepreneurial intention can be defined as the "self-confessed conviction" of anyone that he or she is willing to start a new entrepreneurial activity and plans to carry it out continuously in the future [11]. The entrepreneurial intention has been recognized as a fundamental variable in the study of entrepreneurial behavior because it is a characteristic with a reliable capacity to predict the creation of new businesses in the future [12]. In the field of sustainability, sustainable entrepreneurial intention is a mental state that demonstrates a person’s conviction and commitment to create a new business in the future that generates economic, social, and environmental values [3]; or, in more concrete terms, it is the likelihood that the individual will practice sustainable entrepreneurship in the future [13]. Most research has analyzed students’ entrepreneurial intention in general or students’ social entrepreneurial intention. Recently, Truong et al. [14] stated that studies on sustainable entrepreneurial intention are at an early stage, and there is still an important knowledge gap regarding psychological and social characteristics that affect the personal predisposition to create this type of business.

Complementary, among the psychological characteristics that affect venture development, resilience, and subjective norms have been extensively studied. Psychological resilience can be defined as an individual’s ability to remain strong in the face of a negative situation and to resist that negative situation [15], and subjective norms represent the social pressure perceived by an individual about performing or not performing a certain behavior [16]. Subjective norms are included in Theory of Planned Behavior (TBP) [16] as a contributing factor to general entrepreneurial intention, as well as in other theoretical models that also incorporate social influence and support using the concept of perceptions of desirability, which refers to individuals’ perception of whether their social environment views a behavior as desirable [17, 18]. In addition, resilience can be considered a contextual variable in the Theory of Planned Behavior, since Ajzen states that “Personality traits, intelligence, demographic characteristics, life values, and other variables of this kind are considered background factors in the TPB” [19].

Subjective norms have gained special relevance as an influential factor in entrepreneurial behavior today, due to the high exposure of university students to the opinions of family and friends through social media. Psychological resilience is a quality that has been analyzed in research on business creation during challenging periods and entrepreneurial success [20, 21], and currently it has also become crucial in the education of university students due to the changing conditions of the job market and digital transformation in industries, which demand that professionals be capable of adapting to cope with increased work-life uncertainty. Therefore, while the planned behavior model incorporates other factors such as perceived behavioral control and attitudes towards behavior, this research considers it relevant to analyse the influence of subjective norms and resilience on sustainable entrepreneurial intention, due to the impact of digital business transformation and communication through digital media on university students, as well as the importance of promoting the creation of sustainable businesses in Latin American countries.

Publications have supported the effect of resilience [22] and subjective norms [23] on entrepreneurial intention in general, without specifically deepening the study of the influence of resilience and subjective norms on sustainable entrepreneurial intention. Furthermore, in recent decades, the relevance of perceived social support on the creation of social ventures has been evaluated; in this area, Hockerts [24] supported that this is a factor that favours the creation of social ventures. As in the case of resilience and subjective norms, little is known about the incidence of perceived social support on sustainable entrepreneurial intention in university students. Recently, Acuña-Duran et al. [25] supported the positive influence of perceived social support on entrepreneurial intention in general, without studying sustainable ventures.

On the other hand, some entrepreneurship programs implemented in Latin American universities promote the strengthening of resilience and the obtaining of support from the entrepreneurial ecosystem. However, there is still a low understanding of the effect of resilience and the perception of social pressure and support on the intention to create new sustainable businesses. The knowledge gap in these matters has been argued by Prado et al. [26], who pointed out that while the literature has come a long way in terms of the drivers and motivations underlying value creation in "traditional" entrepreneurs, there remains a significant knowledge gap in terms of the environmental value generation of eco-entrepreneurs. Therefore, given the limited understanding of the psychological and social factors that impact the sustainable entrepreneurial intention of university students, this research raises the following research question: Does resilience and perceived social environment affect entrepreneurial intention associated with sustainable businesses?

This study aims to contributes to improving the understanding of the psychological and social factors that influence the predisposition of university students towards the creation of new sustainable businesses, by providing original information of the role of personal resilience, which is associated with determination and the ability to adapt and recover in the face of adversity [27], and the role of the opinions of relevant people such as family and friends and general social support, in the predisposition towards the creation of new sustainable businesses that promote social welfare and care for the environment. This research aims to enhance comprehension of the factors that foster sustainable entrepreneurial intentions among business and engineering students in Latin America. This is especially pertinent as the curricula of business administration, engineering, and technology students are intricately connected to the creation of solutions aimed at tackling societal challenges [28, 29]. Furthermore, entrepreneurship is acknowledged as a vehicle for personal development in response to evolving conditions in the labor market in Latin America, stemming from shifts in the global and local economy and technological advancements.

## Theoretical frame

### Perceived social support

One of the aspects that influence the development of entrepreneurship is the perceived feasibility of creating businesses, meaning that people perceive that it is more feasible to undertake because of a context that facilitates the creation of businesses in various areas such as governmental, financial, and social support. Thus, several investigations have supported the positive effect of perceived feasibility on entrepreneurial intention [30–32]. Among the domains that affect perceived feasibility, a relevant aspect is the perception of social support; in this sense, it has been recognized that the perception of support from the social environment contributes to shaping a positive perception of the feasibility of creating a business [33]. Perceived social support is a construct that refers to an individual’s perception of the availability of support from his or her social environment, specifically, it is defined as the encouragement and help that an individual expects from his or her close environment to become an entrepreneur [34]. Hockerts [24] supported that greater perceived social support encourages the creation of social businesses, specifically, he argued that people not only consider their own self-efficacy when assessing the feasibility of an action, but also the presence of support systems and networks that will help them achieve the desired outcome.

Research has supported that perceived social support is a predictor of entrepreneurial intention considering the Theory of Planned Behavior (TPB) [25], also that it has a positive effect on entrepreneurial motivation [35] and entrepreneurial attitude orientation [36]. Moreover, previous studies have shown that perceived social support has a positive effect on entrepreneurial intention and behavior by providing the necessary resources and emotional support to pursue their entrepreneurial goals [34]. Perceived social support has also shown a positive association with other relevant entrepreneurship drivers, such as positive attitudes toward business creation and perceived behavioral control [33].

Although some research has recognized that perceived social support is a factor that promotes the development of social businesses [37, 38], few studies have evaluated the relationship between perceived social support and the generation of sustainable ventures that, more broadly, incorporate business initiatives that consider both social welfare and environmental protection through pollutant control, waste minimization and the use of renewable energy, among other aspects. In relation to sustainable entrepreneurship, Truong et al. [14] stated that education and family support, which represent support from the social environment, contribute significantly to strengthening favourable attitudes towards sustainable entrepreneurship, Prado et al. [26] recognized the positive influence of perceived social support on eco-entrepreneurship development, and Muñoz and Dimov [39] argued that social support systems can help sustainable ventures ‐ already created ‐ to maintain or reinforce their high levels of sustainability orientation.

While these studies have not extensively explored the intention of Latin American university students to establish new sustainable businesses in the future, there is evidence that supports the idea that perceived social support plays a positive role in fostering the creation and growth of sustainable entrepreneurship in various ways. Therefore, considering the evidence that supports the impact of perceived social support on overall entrepreneurial development and the development of social ventures, this research proposes that this effect can also be observed when examining the influence of perceived social support on the intention of Latin American university students to engage in sustainable entrepreneurship, which encompasses both social and environmental perspectives. Building upon these notions, the following hypothesis is put forward.

- Hypothesis 1: Perceived social support positively affects sustainable entrepreneurial intention.

Complementarily, the literature regarding entrepreneurial behavior has highlighted that women face special challenges in entrepreneurship, such as lack of credibility, less access to financing, and difficulties to reconcile entrepreneurial activities with their family care responsibilities [40]. Along these lines, Prabawanti and Rusli [41] argued that women need more social support to help them succeed in managing their businesses, and that, consequently, social support becomes an important factor that helps women reduce the occurrence of role conflicts when they perform their activities as entrepreneurs, mothers, and wives simultaneously. This idea is also consistent with the differences in the effect of social support by gender that have been recognized in other areas of study, such as the role of social support in successfully completing university studies [42]. These differences regarding the influence of social support by gender could also be significant when undertaking sustainable businesses, due to the demands of creativity and innovation that are necessary to generate solutions that involve social and environmental benefits. Considering such approaches, this research proposes that the influence of perceived social support on the sustainable entrepreneurial intention of Latin American university students could vary by gender, and the following hypothesis is put forward:

- Hypothesis 2: the influence of perceived support on sustainable entrepreneurial intention is different by gender.

### Subjective norms

Research in recent decades has supported that subjective norms, defined as the perception of social pressure to perform or not to perform a behavior [16], have a significant influence on entrepreneurial attitudes and behaviors. Galleguillos et al. [43] argued that subjective norms measure the value that students assign to the opinion of others close to them, as they value the opinion of family, friends, and classmates when taking an entrepreneurial initiative. Using Ajzen’s model of planned behavior as a reference [16], the influence of subjective norms on attitudes toward entrepreneurship [44] and the positive effect of subjective norms on the entrepreneurial intention of college students [45, 46] have been supported.

Recently, the effect of subjective norms on the intention to create new social businesses and the positive association between subjective norms and sustainable entrepreneurial intention have also been demonstrated [47, 48]. Most of these studies have been conducted in Europe, Asia, and North America and have therefore analyzed responses from study populations with different characteristics from those of Latin American university students. Considering such evidence that tends to support the positive influence of subjective norms on entrepreneurial intention in general and on entrepreneurial intention associated with social business, this research proposes that social pressure from the close environment, that is, pressure from family and friends, should also promote the intention to undertake sustainable business in Latin American university students. Thus, the following hypothesis is proposed.

- Hypothesis 3: Subjective norms positively affect sustainable entrepreneurial intention.

Furthermore, the effect of subjective norms and social support on entrepreneurial attitudes or intentions has been validated [49, 50]. These concepts have common conditions, as they are related to contextual conditions that influence entrepreneurial intention. Likewise, both subjective norms and social support are linked to contextual conditions from complementary perspectives, subjective norms from a close context associated with pressure from friends or family to undertake, and social support from a more global perspective is defined as an individual’s perception of whether their social network adequately supports them or not [51]. In other areas of study, some research has analyzed the combined effect of subjective norms and social support, such as in studies associated with patient decisions in health services [52] and in studies related to online purchases of products [53].

Considering such conceptual relationships, this research proposes that social pressure linked to the opinions of friends or family members, represented by subjective norms, could also have a positive impact on the perception of social support for entrepreneurship since the perception of social support represent the support from external sources as people from social networks. That is to say, individuals who perceive greater social pressure from friends or family to start a business may also perceive that they receive higher social support in initiating an entrepreneurial venture, particularly by perceiving that individuals within their social context promote the creation of their businesses and could also provide assistance in times of difficulties. Thus, this research suggests that social pressure represented in subjective norms could also influence the perception of social support from a broader perspective, and the following hypothesis is proposed:

- Hypothesis 4: Subjective norms positively affect perceived social support.

In addition, significant differences between men and women regarding the influence of subjective norms have been recognized [54, 55] and gender variations in the influence of subjective norms on entrepreneurial attitudes and behaviors have also been supported [56, 57]. Such differences have been explained by discrepancies in gender roles in many societies, particularly in developing societies or societies with traditional cultures. In this line, research has argued that differences in social roles and social stereotypes generate variations in entrepreneurial attitudes and behaviors between men and women [58, 59]. Based on these gender differences that have been supported in previous studies, this research proposes that such variations could also be evidenced by assessing the influence of subjective norms on sustainable entrepreneurial intention and on the perception of social support in Latin American university students. Thus, the following hypotheses are proposed:

- Hypothesis 5: The influence of subjective norms on sustainable entrepreneurial intention is different by gender.
- Hypothesis 6: The influence of subjective norms on perceived social support is different by gender.

### Psychological resilience

Psychological resilience is defined as the mental processes and behaviors that promote an individual’s personal resources to protect him or her against the potential negative effect of stressors [60]. Some studies have supported the positive effect of resilience on entrepreneurial attitudes and behaviors, in that sense, Bullough et al. [61] argued the importance of personal resilience in entrepreneurial intention in danger zones such as war contexts, Santoro et al. [62] supported that personal resilience is positively linked to the perception of entrepreneurial success, and Singh and Chakraborty [63] argued that individual resilience influences the mental wellbeing of entrepreneurs. Although these results constitute valuable evidence highlighting the impacts of psychological resilience on entrepreneurship, such studies have not focused on assessing the entrepreneurial intention of Latin American university students toward sustainable businesses.

In line with such previous evidence, this research proposes that psychological resilience should also positively affect the intention to create new sustainable businesses, in particular, because sustainable entrepreneurship requires a high capacity for transformation and adaptation of resources for the resolution of social and environmental problems [64] and these qualities are consistent with the domains of resilience derived from the literature, since psychological resilience involves the expression of the capacity for determination, resistance, adaptability and recovery [27]. Accordingly, considering the effects of resilience on entrepreneurial behaviors supported in previous studies and the consistency between the characteristics of entrepreneurial sustainability and personal resilience, the following hypothesis is put forward.

- Hypothesis 7: Individual resilience positively affects sustainable entrepreneurial intention.

Moreover, some research has argued the influence of personal resilience on students’ perceptions in diverse domains, such as the effect of resilience on university students’ perceived satisfaction with life [65]. It has also been supported, that resilience contributes to a greater perception of social support in the face of difficult life experiences such as the perception of social support when suffering post-traumatic stress [66] and greater perception of social support in the face of disease recurrence [67]. Little has been studied regarding the effect of personal resilience on perceived social support for business creation, however, based on those studies conducted in other settings, which have shown a positive impact of personal resilience on perceived social support in general life settings and difficult personal situations, this research proposes that psychological resilience could also positively affect the perception of social support related to social entrepreneurship. Accordingly, the following hypothesis is proposed:

- Hypothesis 8: Resilience positively affects perceived social support.

In addition, studies on psychological resilience have evidenced differences in resilience levels between men and women [68–70] and research published in the last two decades has recognized gender differences in entrepreneurial intention, which tends to be higher in men. Tian et al. [71] argued that research has demonstrated gender differences in entrepreneurial intention, that men have demonstrated higher pro-entrepreneurial personality traits than women and women have evidenced lower entrepreneurial intention than men [56, 72]. Regarding perceived social support, some studies have also argued gender discrepancies [73, 74], in particular, research has stated that women tend to reveal more need for social support and to seek more emotional support from their social circles than men, and in this sense, that women tend to perceive greater support from their social environment [75, 76].

In line with evidence that tends to support differences in entrepreneurial attitudes and perceptions by gender, and that suggests that these differences are relevant in cultures with marked distinctions in social roles between men and women, this research proposes that the effect of resilience on sustainable entrepreneurial intention and on perceived social support could be different by gender among Latin American university students. This proposition is also consistent with the marked distinctions in social roles by gender that have been maintained in Latin American countries [77, 78]. Accordingly, the following hypotheses are proposed:

- Hypothesis 9: The influence of resilience on sustainable entrepreneurial intention is different by gender.
- Hypothesis 10: The influence of resilience on perceived social support is different by gender.

## Methodology and methods

### Measurement

An online self-report survey was distributed through the Survey Monkey platform. Emails were sent to students to solicit their responses and a distribution of the survey access link was also conducted to solicit student responses. The online self-report survey entailed students independently completing the survey from their computers or smartphones with an internet connection. This method was selected because, as indicated by Evans and Mathur [79], it offers advantages such as reduced application time and survey costs once an electronic data collection system has been established, simultaneous visualization and tabulation of data during the survey, availability of data in graphic and numerical formats, ease of sending reminders to non-respondents, and ease of importing data into data analysis programs. Furthermore, the online survey has been widely utilized in recent studies on entrepreneurship [80–82].

The survey included a characterization of sustainable entrepreneurship that students had to read before answering each item related to this type of entrepreneurship, in particular, the following description of sustainable entrepreneurship was incorporated in the survey: "Sustainable entrepreneurship is the creation of businesses based on environmentally innovative products and services, which have the potential for substantial success in the market and which contribute to positive social change". The research project was approved by the ethics committee of Universidad Central de Chile (ID:1312022) and the survey incorporated a written informed consent request considering the ethics committee format; only university students who accepted the informed consent request accessed to the online survey questions. The survey was targeted at university students of legal age, in the exceptional case that a student evaluated was not yet 18 years old at the time of answering the survey, no special authorization from the parents or guardians was required since the subject of the survey is entrepreneurship and it is not related to personal or sensitive issues, additionally, the data analysis protected the anonymity of respondents.

The entrepreneurial intention was assessed using the Liñán and Chen [83] scale that includes statements oriented to measure the predisposition to create a new business in the future. The Liñán and Chen [83] entrepreneurial intention scale has been widely used in the last 5 years [84–86] demonstrating adequate reliability in measuring entrepreneurial intention. The measurement of psychological resilience was based on statements from the Brief Resilience Scale (BRS) by Smith et al. [87] derived from the work of Carver [88]; these authors argued that the resilience measures prior to their proposal were very extensive and that BRS is a reliable means to assess resilience in less time. Brief Resilience Scale (BRS) has also demonstrated adequate reliability being used in recent research [89–91] and has been included in several research associated with work activities [92–94] and entrepreneurial resilience [95, 96].

Subjective norms were assessed through the adaptation of the scale published by Liñán and Chen [83], specifically, statements assessing the social pressure from friends and family towards venture creation. This scale associated with subjective norms has demonstrated high reliability and convergence and has been used in several research about entrepreneurial behavior [97–99]; the statement related to the measurement of social pressure from work colleagues was not included in the survey, because a percentage of university students do not work and consequently could not answer this statement.

The measurement of perceived social support towards social entrepreneurship was evaluated through the scale "The Social Entrepreneurial Antecedents Scale (SEAS)" by Hockerts [24], using the social support dimension of this scale that includes statements about the perception of respondents regarding possible access to formal and informal support networks if they created a social enterprise. Such a scale for measuring perceived social support has recently been used in research on entrepreneurship [100, 101]. The selection of this scale was because both social entrepreneurship and sustainable entrepreneurship strive for the advancement of businesses that prioritize the well-being of individuals involved in the production, sale, purchase, and consumption of products. These forms of entrepreneurship are also oriented toward the pursuit of solutions to enhance social welfare. This research incorporated 3 statements from the measurement of perceived social support included in the Entrepreneurial Antecedents Scale (SEAS), because this is the dimension of SEAS that was incorporated in this study.

The items of the scales were translated from English to Spanish and their correct understanding was verified before the final distribution of the online survey. A 7-point Likert scale was used in the measurement, with response options ranging from strongly disagree to strongly agree. Seven levels were selected instead of five as it allows identifying a greater variety of magnitudes [102]. The 7-level frequency scale has been used for decades to assess the frequency of attitudes and perceptions and has also been incorporated in recent studies about sustainable entrepreneurship [103, 104]. The results section presents the reliability and convergence properties achieved in relation to the scales utilized in this study. Gender was asked in the questionnaire and then coded as a dichotomous nominal variable (dummy) in the data preparation stage, the female gender was coded with the number "1" and the male gender with "0".

The overall model, incorporating 433 responses, included gender, the students’ country, and age as control variables, as these factors could influence their sustainable entrepreneurial intention. Previous research has indicated that gender [105], country [106], and age [107] influence the intention to start businesses. Table 1 below presents the statements (observed variables) of each measurement scale. Students under the age of 26 were classified as belonging to the centennial generation according to Sharma [108].

**Table 1:** Statements on measurement scales and response levels.

Latent variables	Statements (observed variables)	Code	Measurement levels
**Resilience**	I tend to bounce back quickly after hard times.	res1	Likert scale from "strongly disagree (1), "disagree" (2), "slightly disagree" (3), "neither disagree nor agree" (4), "slightly agree" (5), "agree" (6) to "strongly agree" (7).
	It does not take me long to recover from a stressful event	res2	
	I usually come through difficult times with little trouble	res3	
**Subjective norms**	My closest family thinks I should create a new business in the future.	sn1	
	My closest friends think that I should create a new business in the future.	sn2	
**Perceived social support**	It is possible to attract investors to create an organisation that wants to solve social problems.	pss1	
	People would support me if I wanted to start an organisation to help socially marginalised people.	pss2	
	If I planned to address a significant social problem people would support me.	pss3	
**Sustainable entrepreneurial intention**	I am ready to do anything to be a sustainable entrepreneur.	ie1	
	My career goal is to become a sustainable entrepreneur.	ie2	
	I will make every effort to start and run my own socially and environmentally sustainable firm.	ie3	
	I am determined to create a socially and environmentally sustainable firm in the future.	ie4	
	I have very seriously thought of starting a socially and environmentally sustainable firm.	ie5	
	I have the firm intention to start a socially and environmentally sustainable firm someday.	ie6	
**Control variables**	Gender	Gen	Woman = 1, Men = 0
	Age cohort	Age	Under 26 years = 1*, Older than 25 years = 0
	Country	Count	Chile = 1, Ecuador = 0

Note: * Students under the age of 26 are categorized as part of the centennial generation.

### Sample

This study has collected responses from business and engineering students in Chile and Ecuador, given the significant importance of training sustainable entrepreneurs for the economic and social advancement of these countries. Additionally, entrepreneurship is a pertinent subject in business and engineering programs, as graduates are anticipated to address societal issues through the establishment of businesses [28, 29].

Convenience sampling was employed, inviting student participation due to resource constraints in this study and the increased expenses associated with random sampling [109, 110], as well as the lower response rates typically observed with online surveys [111]. Several recent studies on students’ entrepreneurial intentions have also utilized convenience sampling [112–114]. The recruitment period for this study spanned from November 15, 2022, to April 10, 2023.

433 students enrolled at Universidad de Las Américas in Chile and Escuela Politécnica in Ecuador answered the questionnaire completely. 291 students enrolled at Universidad de Las Américas in Chile (131 women and 160 men) and 142 students enrolled at Escuela Politécnica in Ecuador (64 women and 78 men). The majority of survey respondents were enrolled in Industrial Engineering (n = 73), Accounting and Auditing (n = 67), and Commercial Engineering (n = 60). The average age of the students was 24.24 years, with a standard deviation of 7.65 years. For male students, the average age was 24.31 years, with a standard deviation of 7.87 years, while for female students, the average age was 24.16 years, with a standard deviation of 7.39 years. Only survey responses that included approval of the informed consent form were included in the analysis. Table 2 outlines the characteristics of the student sample.

**Table 2:** Sample description.

		Women sample		Men sample		Total sample	
		Number	%	Number	%	Number	%
**Age**	< 25 years old	142	72,82%	172	72,57%	314	72,69%
	From 25 to 29 years old	18	9,23%	20	8,44%	38	8,80%
	> 29 years old	35	17,95%	45	18,99%	80	18,52%
**Semester**	From 1 to 4 semester	102	52,31%	133	55,88%	235	54,27%
	From 5 to 12 semester	76	38,97%	93	39,24%	169	39,03%
**Country**	Chile	131	67,18%	160	67,23%	291	60,51%
	Ecuador	64	32,82%	78	32,77%	142	32,79%
**Career**	Industrial Engineering	18	9,23%	55	23,11%	73	16,86%
	Commercial Engineering	25	12,82%	35	14,71%	60	13,86%
	Accounting and Auditing	42	21,54%	25	10,50%	67	15,47%
	Bachelor’s Degree in Administration	37	18,97%	22	9,24%	59	13,63%
	Engineering in Administration	25	12,82%	12	5,04%	37	8,55%
	Production Engineering	18	9,23%	16	6,72%	34	7,85%
	Other careers	30	15,38%	73	30,67%	103	23,79%
	**Total**	195	100,00%	238	100,00%	433	100,00%

**Note:** The table includes the responses correctly recorded, without omitted data. Other careers include students enrolled in programs such as computer engineering and business administration technician. In Latin America, programs in management and engineering typically range from 4 to 6 years, or 8 to 12 semesters.

Despite the limitations of convenience sampling, the sample demonstrates a distribution of gender, age, and income that aligns with the demographic characteristics of students in Chile and Ecuador. The higher representation of men in fields such as industrial engineering and other engineering disciplines is consistent with the lower participation of women in science, technology, engineering, and mathematics (STEM) careers in Latin America [115].

Students enrolled at Universidad de Las Américas in Chile and Escuela Politécnica in Ecuador predominantly come from families with medium and lower-middle incomes, reflecting the income profiles of the undergraduate student populations in both countries. This trend can be attributed to the increased availability of public and private funding for university access over the past two decades, leading to a significant rise in university enrolment across various income brackets in these nations [116, 117].

Furthermore, the age distribution of the students in the sample aligns with that of university students in Chile and Ecuador, with the majority of surveyed students being under 30 years old. Data from the Higher Education Information Service of Chile [118] indicates that in Chile, undergraduate students under 25 years old constitute 65.1% of the total, those aged 25 to 29 make up 15.9%, individuals aged 29 to 39 represent 12.9%, and those over 40 comprise 6% of the student population. Similarly, Stefos [119] observed that in Ecuador, students under 30 years old make up 90.95% of the total, while students aged 30 or older represent 9.05%.

### Statistical analysis

This research has used PLS-SEM to evaluate the relationship between the proposed latent variables and the acceptance or rejection of hypotheses. This is one of the most widely used multivariate data analysis methods by business and social science researchers [120] and also in other areas such as marketing, hospitality and tourism, and human resources [121, 122]. In the field of entrepreneurial behavior, PLS-SEM models have been widely used in the last 2 years [123–126]. One of the reasons for the wide acceptance of PLS-SEM is that it allows to simultaneously analyse the relationships between observed and latent variables in a complex model and to perform multiple robustness assessments considering the measurement error that is inherent in the evaluation of abstract concepts [127, 128]. Additionally, this is a more flexible modeling methodology, as it does not require rigorous parametric assumptions, mainly associated with the distribution of the data [129]; in this sense, Wolf [130] stated that PLS-SEM does not require the conditions of traditional covariance structural equation modeling (CB-SEM) related to statistical distributions (normality of the data).

PLS-SEM assesses the statistical significance of factor loadings and standardized regression coefficients between latent variables. The explanatory power of PLS-SEM models is evaluated using metrics such as the average R2 parameter, Goodness of Fit (GOF), Q2, and F2, which are commonly employed to gauge the predictive capability of PLS-SEM models in entrepreneurship studies [131, 132]. The R2 parameter serves as an indicator of the predictive performance of the structural model [133]. GOF, calculated as the geometric mean of the average communality [134], assesses the overall model fit of the PLS-SEM model [135]. Q2 represents the predictive relevance of the model, with Stone and Geisser proposing it as a measure of the model’s out-of-sample predictive ability [136, 137]. Similar to the R2 parameter, the F2 parameter represents the ratio between the explained and unexplained variance (F2 = R2 / (1- R2)) [138].

A reliability and convergence analysis of the constructs (latent variables) has been conducted, through the calculation of the standardized loadings of the observed variables, the Dillon-Goldstein parameter, and Cronbach’s alpha parameter, which is a measure of internal consistency and is expressed as a number between 0 and 1 [139]. Dillon-Goldstein’s ρ and Cronbach’s α indicate the reliability of the summed scores. While Cronbach’s α is based on the variance-covariance matrix of the indicator, Dillon-Goldstein’s ρ is based on the factor loadings. In addition, the Average Variance Extracted (AVE) parameter was calculated, which is defined as the average variance extracted from the items of a particular construct, that is, the AVE values show how much the construct explains the variance of its items [140]. Furthermore, the Fornell-Larcker criterion [141] was utilized to assess discriminant validity, which stipulates that the square root of the AVE of each construct must exceed the correlation coefficients between two constructs. Discriminant validity pertains to the extent to which a construct differs from other constructs [142].

Gender comparison analysis was carried out using multi-group analysis (MGA) in PLS-SEM. MGA is a widely employed approach for evaluating significant differences between subgroups of the sample [143, 144]. It encompasses a set of advanced techniques typically applied when researchers seek to examine disparities between categorical variables (e.g., gender or countries) or continuous variables that can be categorized [145, 146]. PLS-MGA allows researchers to assess significant differences between the structural paths of multiple groups [147]. This methodology was chosen as one of the study’s objectives is to evaluate gender differences, and recent studies on entrepreneurial intention employing PLS-SEM have utilized MGA to test distinctions between demographic groups [148–150]. The statistical analysis was conducted using the plssem package in STATA version 17.

## Results

### Descriptive analysis

Table 3 displays the central tendency of the observed variables through the calculation of the arithmetic mean and the median, as well as the dispersion of the observed variables through the calculation of the standard deviation. It is acknowledged that the median reaches level 5 in all observed variables, except for the statements "I have very seriously thought of starting a socially and environmentally sustainable firm" (ie5) and "I have the firm intention to start a socially and environmentally sustainable firm someday" (ie6), which show a mean of 4. Additionally, the arithmetic mean of all observed variables was greater than 4.0 (the midpoint on the Likert scale of 7 points), which suggests that students tended to agree with statements related to psychological resilience, subjective norms, perceived social support, and entrepreneurial intention. The standard deviation of the observed variables falls within the range of 1.279 to 1.904, and the coefficient of variation falls within the range of 26% to 39%.

**Table 3:** Central tendency and dispersion analysis.

	res1	res2	res3	ns1	ns2	aes1	aes2	aes3	ie1	ie2	ie3	ie4	ie5	ie6
**Mean**	5.14	4.92	5.03	4.83	5.14	4.73	4.82	4.92	5.21	4.67	4.89	4.62	4.43	4.36
**Median**	5.00	5.00	5.00	5.00	5.00	5.00	5.00	5.00	5.00	5.00	5.00	5.00	4.00	4.00
**Standard deviation**	1.43	1.57	1.48	1.90	1.68	1.40	1.28	1.26	1.34	1.54	1.43	1.55	1.66	1.63
**Coefficient of variation**	29%	32%	30%	39%	33%	30%	27%	26%	26%	33%	29%	34%	37%	37%

### Reliability and convergent validity

Acceptable values for Cronbach’s α and Dillon-Goldstein’s ρ (DG) are typically above 0.70 [151]. As depicted in Table 4, all scales incorporated in the model demonstrated Cronbach’s alpha reliability levels ranging from 0.797 to 0.948, and Dillon-Goldstein parameters exceeding 0.80 for all scales. Moreover, standardized loadings surpassing 0.50 and average variance extracted (AVE) values above 0.50 are deemed acceptable [152, 153]. Table 4 illustrates standardized factor loadings surpassing 0.60 and AVE values exceeding 0.70 for the scales included in the measurement instrument, for all constructs. Hence, these parameters substantiate the adequate levels of reliability and convergence of the measurement scales.

**Table 4:** Factor loadings, Cronbach’s alpha and AVE.

	Code	Standardized factor loadings				Cronbach α	Dillon-Goldstein ρ	AVE
**Resilience**	res1	0.934				0.804	0.881	0.715
	res2	0.698						
	res3	0.886						
**Subjective norms**	sn1		0.938			0.869	0.938	0.884
	sn2		0.942					
**Perceived social support**	pss1			0.741		0.797	0.882	0.715
	pss2			0.894				
	pss3			0.892				
**Sustainable Entrepreneurial intention**	ie1				0.782	0.948	0.959	0.797
	ie2				0.908			
	ie3				0.909			
	ie4				0.928			
	ie5				0.917			
	ie6				0.905			

Table 5 contrasts the correlations of the latent variables with the square root of the AVE coefficients. The results support the adequate discriminant validity of the latent variables, in line with the criterion set by Fornell and Larcker [141], which stipulates that the square root of the AVE coefficients must exceed the correlation coefficients between the latent variables.

**Table 5:** Discriminant validity ‐ interfactor correlation vs. squared root of AVE.

	Resilience	Subjective norms	Perceived social support	Sustainable Entrepreneurial intention
**Resilience**	*0*.*846*			
**Subjective norms**	0.304	*0*.*940*		
**Perceived social support**	0.325	0.340	*0*.*846*	
**Sustainable Entrepreneurial intention**	0.220	0.411	0.443	*0*.*893*

Note: representation of the results of the discriminant analysis conducted according to the criterion of Fornell and Larcker. The diagonals represent the square root of the AVE, while the off-diagonals represent the correlations.

### PLS-SEM model

The predictive power and fit of the PLS-SEM model are presented in Table 6. Absolute GOF values greater than 0.36 indicate a good fit for the model [154]. R2 values exceeding 0.20 signify an acceptable level of explanatory power, although weak [140]. Q2 values of 0.02, 0.15, and 0.35 indicate the predictive relevance of an exogenous construct as small, medium, or large for a specific endogenous construct [138]. The obtained parameters include R2 values above 0.20, absolute GOF values surpassing 0.36, Q2 values greater than 0.35, and F2 values exceeding 0.150. These metrics indicate an acceptable level of explanatory power and goodness of fit for the PLS-SEM model.

**Table 6:** Structural model–predictive power and fit.

	Number of observations	R2	Absolute GOF	Q2	F2
Total	433	0.225	0.417	0.403	0.290
Men	238	0.250	0.441	0.452	0.334
Women	195	0.212	0.405	0.384	0.270

Table 7 presents the analysis of all responses (n = 433) assessing the acceptance or rejection of hypotheses 1 to 8 based on the obtained evidence. The standardized coefficient associated with the Resilience → Perceived social support trajectory was positive and significant (β = 0.244, p = 0.000), as were the standardized coefficients associated with the trajectory of Subjective norms → Perceived social support (β = 0.265, p = 0.000) and Subjective norms → Sustainable entrepreneurial intention (β = 0.279, p = 0.000). Similarly, the positive and significant effect of Perceived social support on sustainable entrepreneurial intention has been demonstrated with 99% confidence (β = 0.342, p = 0.000). The trajectory associated with Resilience → Sustainable entrepreneurial intention was not statistically significant (β = 0.027, p = 0.621).

**Table 7:** Structural equation models ‐ standardized coefficients.

	β	p-value	SE	IC (95%)	VIF	f2	Hypothesis
Perceived SS → Sustainable IE	0.342	**0.000**	0.051	(0.241; 0.443)	1.289	0.058	H1: Accepted
S. Norms → Sustainable IE	0.279	**0.000**	0.049	(0.181; 0.337)	1.231	0.040	H3: Accepted
S. Norms → Perceived SS	0.265	**0.000**	0.050	(0.165; 0.365)	1.102	0.041	H4: Accepted
Resilience → Sustainable IE	0.027	**0.621**	0.055	(-0.083; 0.137)	1.251	0.001	H7: Rejected
Resilience → Perceived SS	0.244	**0.000**	0.053	(0.137; 0.351)	1.102	0.035	H8: Accepted
*Control variables*							
Country → Sustainable IE	-0.032	0.510	0.048	(-0.129; 0.065)	1.217	0.001	
Age cohort → Sustainable IE	-0.069	0.128	0.045	(-0.160; 0.022)	1.146	0.003	
Gender → Sustainable IE	0.061	0.149	0.042	(-0.023; 0.145)	1.051	0.002	

Table 7 includes values of standard error, confidence intervals, and variance inflation factors (VIF), all of which are below 2.0, indicating no multicollinearity issues [155]. In addition, the analysis includes Cohen’s f2 values, which are calculated by observing the change in R2 when a specific construct is removed from the model. According to Cohen [156, 157], f2 values of 0.02, 0.15, and 0.35 represent small, medium, and large effects, respectively. The coefficients and p-values of the control variables are presented, none of which showed a statistically significant effect on sustainable entrepreneurial intention. Finally, Table 6 presents the acceptance or rejection of the hypotheses based on this evidence.

**Fig 1:**
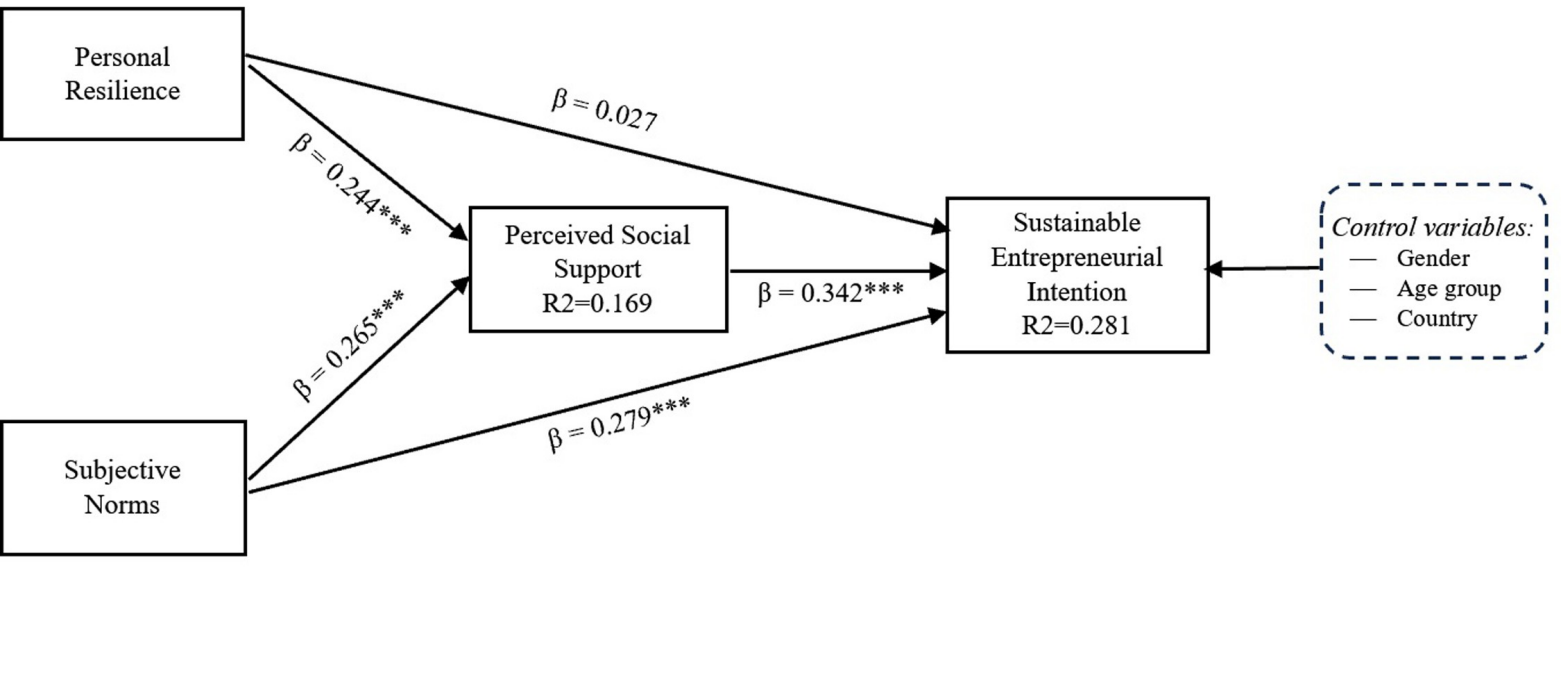
Results of global PLS SEM model.

Fig 1 below represents the relationships between latent variables included in the PLS-SEM model evaluated in this research, these relationships are associated with the previously proposed hypotheses.

When conducting the analysis in the group of men (n = 238) and women (n = 195), similar results were obtained regarding the significance of the standardized coefficients, since like in the total sample, all standardized coefficients were significant with 95% confidence (p <0.050), except for the coefficient associated with the Resilience → Sustainable entrepreneurial intention trajectory. Table 8 shows these standardized coefficients between latent variables by gender, evaluating significant differences between them through a hypothesis test based on Z-distribution parameters according to MGA. The results have only supported significant gender differences in the influence of subjective norms on sustainable entrepreneurial intention with 95% confidence (p = 0.046). Table 8 also presents the acceptance or rejection of the hypotheses associated with gender differences according to the p-values obtained.

**Table 8:** Comparison of standardized coefficients by gender.

	β Women	p-value Women	β Men	p-value Men	β Difference	p-value Difference	Hypothesis
Perceived SS → Sustainable IE	0.288	0.002	0.402	0.000	0.114	0.247	H2: Rejected
S. Norms → Sustainable IE	0.187	0.015	0.373	0.000	0.187	0.049	H5: Accepted
S. Norms → Perceived SS	0.351	0.000	0.188	0.001	0.163	0.125	H6: Rejected
Resilience → Sustainable IE	0.111	0.182	-0.058	0.382	0.170	0.108	H9: Rejected
Resilience → Perceived SS	0.230	0.003	0.269	0.000	0.039	0.808	H10: Rejected
*Control variables*							
Country → Sustainable IE	-0.034	0.675	-0.040	0.499	0.006	0.876	
Age cohort → Sustainable IE	-0.079	0.248	-0.048	0.415	0.031	0.786	

## Discussion

### Theorical implications

The purpose of this research was to evaluate the effect of psychological resilience and subjective norms on perceived social support, as well as to estimate the influence of these three psychological variables on sustainable entrepreneurial intention. The findings summarized in Table 7 have supported a positive influence of psychological resilience and subjective norms on perceived social support, as well as a significant positive influence of subjective norms and perceived social support on sustainable entrepreneurial intention. The findings summarized in Table 8 have supported gender differences in the influence of subjective norms on sustainable entrepreneurial intention, which has been greater in male students at 95% confidence.

The results related to subjective norms and perceived social support are framed within theoretical models that have explained entrepreneurial behavior. These models argue that social pressure and support impact the intention to start new businesses. The Theory of Planned Behavior by Ajzen [16] includes subjective norms as a determinant factor of entrepreneurial behavior, this concept represents the perceived social pressure to engage in the behavior and the motivation to comply with those norms or expectations [158]. Research has widely supported the effect of subjective norms on entrepreneurial intentions [45, 159, 160]. In this line, van Gelderen et al. [161] stated that individuals may seek advice and support from those in their social environment, and as a result, the opinions of significant others would influence how individuals would or would not engage in entrepreneurship.

The Entrepreneurial Event Model proposed by Shapero and Sokol [17] suggests that two important determinants of entrepreneurial intention are the perceived influence of the social environment, which is part of the "perceptions of desirability" factor, and the perception of support regarding financing, mentors, and partners, which is integrated into the "perceptions of feasibility" factor. The perceptions of desirability align with the concept of subjective norms, as the influence of friends and family is part of the social environment. The perceptions of feasibility align with the concept of perceived social support, as both terms relate to social support. The positive effect of perceptions of feasibility and perceptions of desirability on entrepreneurial intention has been widely supported in previous studies [162–164]. Similarly, the Entrepreneurial Intention Model proposed by Liñán [18] suggests that subjective norms fall within the realm of "perceived desirability" and exert a significant influence on entrepreneurial intention in general. Previous research has also supported the influence of "perceived desirability" on entrepreneurial intention using the Entrepreneurial Intention Model as the theoretical framework [165, 166].

However, this study enhances the understanding of the impact of psychological and social conditions on sustainable entrepreneurial intention by presenting new evidence that complements previous studies grounded in these theoretical frameworks. Specifically, this research demonstrates that personal resilience, in conjunction with subjective norms, positively influences perceived social support, which in turn plays a significant role in the manifestation of entrepreneurial intention. Furthermore, it focuses on investigating the effect of these factors on the intention to initiate new sustainable businesses that prioritize environmental protection and social well-being, given the increasing importance attributed to these ventures in recent years. In addition, it examines university students in Latin America due to the growing significance of entrepreneurship and sustainability in higher education within this region.

According to the results of this study, personal resilience affects perceived social support, meaning that students who expressed greater resilience and adaptability in the face of problems also tended to have a higher perception of support for the creation of social ventures. This result is noteworthy as it demonstrates that the ability to recover and adapt in the face of challenges is related to a better perception of support from the environment, meaning that more resilient individuals appreciate social support in a more effective manner when initiating social ventures. Moreover, these findings are aligned with evidence obtained in other areas of study. For example, there is a positive relationship between resilience and perceived social support when studying healthcare personnel [167] and individuals with serious illnesses [168]. Furthermore, research has supported the positive association between resilience and social support when analysing school and university students [169, 170], as well as when examining emergency situations due to the COVID-19 pandemic [171, 172].

The positive effect of subjective norms on perceived social support and sustainable entrepreneurial intention, supported in this study, highlights that the pressure of close individuals such as friends and family positively influences both the perception of support from the social environment and the intention to undertake. This result is consistent as both concepts are linked to contextual conditions in a complementary way. Subjective norms are associated with pressure from friends or family in a close context, while social support is considered from a broader perspective, encompassing perceptions of support from the general social environment [51]. Furthermore, these results emphasize the need to generate social contexts that incentivize the creation of sustainable businesses in Latin American universities, through focused initiatives such as the creation of entrepreneurship seedbeds that strengthen support networks and promote favourable opinions from close individuals regarding sustainable entrepreneurship.

The positive effect of perceived social support on sustainable entrepreneurial intention supported in this research is also consistent with studies that have argued the importance of the perception of environmental support to promote perceived feasibility and thus the creation of new businesses [173, 174]. In this regard, Cavazos et al. [175] highlighted that the lack of social support can be considered an inhibitor of entrepreneurial activity among university students and that the absence of perceived social support may be because university students do not identify the existence of systems and/or support networks to develop their potential businesses. These results have been replicated in the field of social entrepreneurship, as de Sousa-Filho et al. [176] showed that perceived social support positively influences the intention to create social businesses. Additionally, in the field of sustainable entrepreneurship, it has been argued that social support systems can help already established sustainable ventures to maintain or reinforce their high levels of organizational sustainability orientation [39].

This study has found support for gender differences in the influence of subjective norms on sustainable entrepreneurial intention, with a greater effect observed among male students at a 95% confidence level. This result is consistent with previous research that has identified discrepancies between men and women in terms of their perception of subjective norms [54, 55] and entrepreneurial intention [56, 72]. Moreover, gender discrepancies associated with the effect of subjective norms on entrepreneurial intention is in line with studies that have shown discrepancies in the effect of social pressure between men and women in other areas, such as the perception of success at work. In this sense, McColl-Kennedy and Dann [177] argued that women’s self-perception of work success is more subjective and related to feelings, while men may perceive greater social pressure and associate success with more concrete aspects such as obtaining higher income or social status. Similarly, this result could be explained by gender role differences in families, as such discrepancies in family roles between men and women continue to be relevant in Latin America [178, 179]; Thus, the opinion of friends and family associated with subjective norms could have a greater effect on sustainable entrepreneurial intention in men, due to the fact that the male gender is still more associated with providing family income in this region.

#### Practical implications

The results have important practical implications for university administrators such as deans or program directors. Those responsible for the training of professionals may consider the findings of this study to strengthen the sustainable entrepreneurial intention of their business and engineering students. Psychological resilience, which promotes personal adaptation to overcome difficulties, could be strengthened through university initiatives such as strategic partnerships between universities and public or private support networks. Such initiatives should develop students’ capacities to overcome challenges and adapt to difficulties.

Additionally, perceived social support could be strengthened through the implementation of programs or projects that foster university-community engagement. These initiatives should bring together students with active entrepreneurs or individuals working in entrepreneurship ecosystem organizations, such as business incubators or government institutions dedicated to supporting entrepreneurship. While there is little evidence on the effect of these intervention programs on perceived social support for entrepreneurship, some research has demonstrated the benefits of programs to strengthen social support in domains other than entrepreneurship. Along these lines, the benefits of social support programs have been shown in the psychological recovery of older adults who have faced traumatic events [180] and the benefits of programs aimed at reducing the stress of medical personnel [181].

The findings of this study can also serve as a guide for public policies aimed at strengthening sustainable entrepreneurship in Latin American countries. Policymakers could implement support programs for entrepreneurship aimed at enhancing perceptions of obtaining support and attracting investments for the development of ventures that address social problems. Governments could also design and implement programs to promote sustainable entrepreneurship that encourage family or community collaboration, with the purpose of fostering social influence that, in turn, promotes sustainable entrepreneurship. Additionally, governments could fund programs to support the development of sustainable businesses that focus on individuals who have faced adversity and demonstrated resilience and adaptability, such as those facing health difficulties or who have immigrated due to political and economic issues in their countries of origin.

## Conclusions

This research provides new evidence about the psychological and social factors that influence Latin American university students’ sustainable entrepreneurial intention. The results have supported the effect of personal resilience and social pressure, represented in the concept of subjective norms, on the perception of social support and the intention to create new sustainable businesses. Likewise, the high influence of perceived social support on sustainable entrepreneurial intention has been highlighted, being observed in the higher path coefficients of PLS-SEM models. A greater influence of subjective norms on entrepreneurial intention was also evidenced in male students.

The results of this research will allow the actors involved in higher education to propose strategies that foster the sustainable entrepreneurial intention of university students who are potentially involved in the development of new socially and environmentally sustainable ventures. Besides, the evidence obtained in this research can be useful for the development of Latin American countries through the strengthening of sustainable entrepreneurship, since the countries in this region have an infinity of natural resources, but they are also immersed in a series of economic, social, and political problems that could be overcome through sustainable entrepreneurship.

### Limitations and future research

This research analyses the responses of business administration and engineering and technology students enrolled in universities in Chile and Ecuador. Future research should analyse the sustainable entrepreneurial intention of students enrolled in other Latin American countries to corroborate the validity of the results obtained in the region in general, as well as to evaluate the relationships between latent variables recognized in this research in countries of other continents, such as Africa, Europe, and Oceania. On the other hand, the convenience sample used in the research limits the generalizability of the results, although it has been supported that the gender distribution of the sample was consistent with the characteristics of students in Latin America. Finally, future studies should explore the relationship between resilience, subjective norms and perceived social support, and other important variables in the field of sustainable entrepreneurial behavior, such as their relationship with perceived entrepreneurial success or with the expression of innovative behaviors to create sustainable businesses.
